# Supplementary material for: European Prevalence of Polypoidal Choroidal Vasculopathy: A Systematic Review, Meta-Analysis, and Forecasting Study
Source: J Clin Med. 2022 Aug 16;11(16):4766. doi: 10.3390/jcm11164766 (PMC9410106; doi:10.3390/jcm11164766)
Supplement: Supplementary file 1 [file jcm-11-04766-s001.zip › Supplementary Data S1.pdf]

Supplementary Data S1: Population statistics and forecasting of current and future number of in-dividuals aged ≤ 64 years, 65–74 years, and ≥ 75 years in European countries

Year	2022	2025	2030	2035	2040
Population ≤ 64 years					
Belgium	9296099	9243942	9101966	8983573	8903825
Bulgaria	5320670	5150069	4886086	4637655	4376466
Czechia	8547991	8513870	8390853	8242589	7993344
Denmark	4639961	4633136	4606341	4562212	4535279
Germany	64829262	64163971	62260395	60471999	59993740
Estonia	1053318	1035637	1005145	980099	952797
Ireland	4336840	4434690	4536512	4618041	4665196
Greece	8193455	7990612	7641550	7251592	6894486
Spain	38219366	37992440	37155111	36132179	35016825
France	53260392	52964353	52326993	51730451	51168091
Croatia	3125862	3021787	2867937	2730893	2612246
Italy	45898268	45238370	43741742	41985184	40381279
Cyprus	749772	760794	775038	789430	801644
Latvia	1480330	1406173	1286543	1187357	1098371
Lithuania	2212617	2114270	1934755	1783191	1651212
Luxembourg	543663	556284	567533	572089	575155
Hungary	7723091	7634790	7539678	7375069	7145308
Malta	429020	446348	465226	482727	494130
Netherlands	14020086	13958637	13748319	13534158	13403555
Austria	7197345	7169033	7048059	6903498	6835577
Poland	30478644	29651766	28621800	27732783	26648786
Portugal	7931722	7767302	7441100	7131028	6791367
Romania	15194317	14609728	13927082	12988730	12152675
Slovenia	1664777	1642451	1591576	1542653	1502647
Slovakia	4502070	4429813	4302258	4181099	4019733
Finland	4256985	4200930	4096729	4010070	3966028
Sweden	8375806	8537070	8735384	8873838	9030598
Iceland	322901	333053	344269	355527	365884
Liechtenstein	31544	31453	31064	30644	30503
Norway	4456646	4509433	4588284	4635680	4682487
Switzerland	6986050	7025193	7055162	7073260	7144276
United Kingdom	54718597	54730950	54210505	53632524	53470997

	2022	2025	2030	2035	2040
Population 65–74 years					
Belgium	1208260	1260433	1364733	1393462	1358056
Bulgaria	875761	844667	805452	787190	818708
Czechia	1288134	1224630	1148242	1154108	1302064
Denmark	629613	624090	665497	713960	714682
Germany	9163017	9797087	11076771	11756153	10453673
Estonia	147653	150846	153262	148744	151192
Ireland	432167	460356	513278	562187	621326
Greece	1197694	1228199	1288240	1360225	1406771
Spain	4827190	5162722	5916192	6520039	6944153
France	7608960	7629237	7848856	8058316	8189280
Croatia	501519	514845	510306	486063	461110
Italy	6975930	7097786	7955849	8797829	9000317
Cyprus	87626	91076	96538	96394	94438
Latvia	209335	217120	226402	213468	202448
Lithuania	292999	318652	353081	344686	321690
Luxembourg	53144	58313	69276	79202	83376
Hungary	1190069	1168220	1038374	1025077	1161329
Malta	57849	58622	60950	59861	62912
Netherlands	1962901	1995735	2162198	2271010	2201465
Austria	893156	945418	1117153	1222237	1167336
Poland	4537949	4724801	4406187	3898990	4030920
Portugal	1202596	1241225	1315572	1356489	1389962
Romania	2234769	2246628	1983816	2065088	2323949
Slovenia	250210	260893	265140	267797	266581
Slovakia	605051	630263	622710	597074	629764
Finland	703376	676761	666433	653724	603687
Sweden	1078624	1056969	1107127	1199170	1246479
Iceland	32815	35879	39870	41488	43172
Liechtenstein	4287	4575	5273	5703	5577
Norway	541053	553800	590872	642312	681001
Switzerland	849206	893165	1031599	1134342	1123297
United Kingdom	6675601	6827249	7687973	8254738	8036869

	2022	2025	2030	2035	2040
Population ≥ 75 years					
Belgium	1076527	1156831	1291291	1458785	1633000
Bulgaria	647478	695652	758758	799204	821545
Czechia	922246	1051306	1223079	1297739	1329994
Denmark	566213	627049	691740	742644	805542
Germany	9354025	9521249	10116531	11090518	12731013
Estonia	127596	135957	150028	165200	177566
Ireland	331981	377884	454600	534143	618018
Greece	1240890	1291385	1373410	1492805	1609541
Spain	4819158	5155457	5675096	6458651	7416116
France	6669779	7443218	8573551	9565554	10445038
Croatia	379458	399877	449846	504076	539131
Italy	7301928	7752373	8244921	8926969	9993410
Cyprus	66230	76425	91278	104990	116776
Latvia	187942	192257	199801	218002	235289
Lithuania	272248	274993	287717	324436	366796
Luxembourg	43458	47767	55913	66813	80606
Hungary	830521	894210	1040968	1132684	1134502
Malta	43987	52456	62515	71626	77868
Netherlands	1584679	1796683	2059367	2314383	2580772
Austria	857166	914557	983789	1106973	1289450
Poland	2806289	3190681	3990466	4737555	4981950
Portugal	1145928	1212074	1332466	1461477	1605303
Romania	1554421	1651191	1897102	2115470	2099563
Slovenia	195374	211259	249600	284204	312394
Slovakia	359315	407815	515762	606439	662942
Finland	576003	659750	756136	817177	856428
Sweden	1047158	1152847	1256522	1332415	1416296
Iceland	23587	26704	32851	40116	46815
Liechtenstein	3351	3893	4679	5514	6451
Norway	447988	505310	586163	663344	732464
Switzerland	824008	893116	989885	1110952	1275342
United Kingdom	6201626	6746733	7308719	7975723	8910517
