# Supplementary material for: European Prevalence of Polypoidal Choroidal Vasculopathy: A Systematic Review, Meta-Analysis, and Forecasting Study
Source: J Clin Med. 2022 Aug 16;11(16):4766. doi: 10.3390/jcm11164766 (PMC9410106; doi:10.3390/jcm11164766)
Supplement: Supplementary file 1 [file jcm-11-04766-s001.zip › Supplementary Data S2.pdf]

Supplementary Data S2: Estimated current and future number of patients with neovascular age-related macular degeneration in European countries.

Year	2022	2025	2030	2035	2040
Population ≤ 64 years					
Belgium	9296	9244	9102	8984	8904
Bulgaria	5321	5150	4886	4638	4376
Czechia	8548	8514	8391	8243	7993
Denmark	4640	4633	4606	4562	4535
Germany	64829	64164	62260	60472	59994
Estonia	1053	1036	1005	980	953
Ireland	4337	4435	4537	4618	4665
Greece	8193	7991	7642	7252	6894
Spain	38219	37992	37155	36132	35017
France	53260	52964	52327	51730	51168
Croatia	3126	3022	2868	2731	2612
Italy	45898	45238	43742	41985	40381
Cyprus	750	761	775	789	802
Latvia	1480	1406	1287	1187	1098
Lithuania	2213	2114	1935	1783	1651
Luxembourg	544	556	568	572	575
Hungary	7723	7635	7540	7375	7145
Malta	429	446	465	483	494
Netherlands	14020	13959	13748	13534	13404
Austria	7197	7169	7048	6903	6836
Poland	30479	29652	28622	27733	26649
Portugal	7932	7767	7441	7131	6791
Romania	15194	14610	13927	12989	12153
Slovenia	1665	1642	1592	1543	1503
Slovakia	4502	4430	4302	4181	4020
Finland	4257	4201	4097	4010	3966
Sweden	8376	8537	8735	8874	9031
Iceland	323	333	344	356	366
Liechtenstein	32	31	31	31	31
Norway	4457	4509	4588	4636	4682
Switzerland	6986	7025	7055	7073	7144
United Kingdom	54719	54731	54211	53633	53471
Total	419997	415898	406831	397142	389305

	2022	2025	2030	2035	2040
Population 65–74 years					
Belgium	9666	10083	10918	11148	10864
Bulgaria	7006	6757	6444	6298	6550
Czechia	10305	9797	9186	9233	10417
Denmark	5037	4993	5324	5712	5717
Germany	73304	78377	88614	94049	83629
Estonia	1181	1207	1226	1190	1210
Ireland	3457	3683	4106	4497	4971
Greece	9582	9826	10306	10882	11254
Spain	38618	41302	47330	52160	55553
France	60872	61034	62791	64467	65514
Croatia	4012	4119	4082	3889	3689
Italy	55807	56782	63647	70383	72003
Cyprus	701	729	772	771	756
Latvia	1675	1737	1811	1708	1620
Lithuania	2344	2549	2825	2757	2574
Luxembourg	425	467	554	634	667
Hungary	9521	9346	8307	8201	9291
Malta	463	469	488	479	503
Netherlands	15703	15966	17298	18168	17612
Austria	7145	7563	8937	9778	9339
Poland	36304	37798	35249	31192	32247
Portugal	9621	9930	10525	10852	11120
Romania	17878	17973	15871	16521	18592
Slovenia	2002	2087	2121	2142	2133
Slovakia	4840	5042	4982	4777	5038
Finland	5627	5414	5331	5230	4829
Sweden	8629	8456	8857	9593	9972
Iceland	263	287	319	332	345
Liechtenstein	34	37	42	46	45
Norway	4328	4430	4727	5138	5448
Switzerland	6794	7145	8253	9075	8986
United Kingdom	53405	54618	61504	66038	64295
Total	466548	480002	512746	537337	536781

	2022	2025	2030	2035	2040
Population ≥ 75 years					
Belgium	35525	38175	42613	48140	53889
Bulgaria	21367	22957	25039	26374	27111
Czechia	30434	34693	40362	42825	43890
Denmark	18685	20693	22827	24507	26583
Germany	308683	314201	333846	365987	420123
Estonia	4211	4487	4951	5452	5860
Ireland	10955	12470	15002	17627	20395
Greece	40949	42616	45323	49263	53115
Spain	159032	170130	187278	213135	244732
France	220103	245626	282927	315663	344686
Croatia	12522	13196	14845	16635	17791
Italy	240964	255828	272082	294590	329783
Cyprus	2186	2522	3012	3465	3854
Latvia	6202	6344	6593	7194	7765
Lithuania	8984	9075	9495	10706	12104
Luxembourg	1434	1576	1845	2205	2660
Hungary	27407	29509	34352	37379	37439
Malta	1452	1731	2063	2364	2570
Netherlands	52294	59291	67959	76375	85165
Austria	28286	30180	32465	36530	42552
Poland	92608	105292	131685	156339	164404
Portugal	37816	39998	43971	48229	52975
Romania	51296	54489	62604	69811	69286
Slovenia	6447	6972	8237	9379	10309
Slovakia	11857	13458	17020	20012	21877
Finland	19008	21772	24952	26967	28262
Sweden	34556	38044	41465	43970	46738
Iceland	778	881	1084	1324	1545
Liechtenstein	111	128	154	182	213
Norway	14784	16675	19343	21890	24171
Switzerland	27192	29473	32666	36661	42086
United Kingdom	204654	222642	241188	263199	294047
Total	1732782	1865126	2069250	2294377	2537978

	2022	2025	2030	2035	2040
Total					
Belgium	54488	57503	62632	68271	73657
Bulgaria	33694	34864	36369	37309	38037
Czechia	49287	53004	57938	60301	62300
Denmark	28362	30318	32758	34781	36836
Germany	446816	456742	484720	520508	563747
Estonia	6445	6729	7182	7622	8022
Ireland	18750	20588	23645	26742	30030
Greece	58724	60432	63270	67396	71264
Spain	235869	249424	271763	301428	335302
France	334235	359624	398045	431860	461369
Croatia	19660	20336	21795	23254	24092
Italy	342669	357849	379471	406958	442166
Cyprus	3636	4011	4560	5025	5411
Latvia	9357	9488	9691	10089	10482
Lithuania	13541	13738	14254	15247	16329
Luxembourg	2403	2599	2967	3411	3902
Hungary	44651	46489	50199	52954	53875
Malta	2343	2646	3016	3325	3567
Netherlands	82018	89215	99005	108077	116181
Austria	42629	44913	48450	53212	58726
Poland	159390	172743	195557	215264	223300
Portugal	55368	57696	61937	66212	70886
Romania	84368	87072	92402	99320	100030
Slovenia	10114	10701	11949	13064	13944
Slovakia	21200	22930	26304	28970	30935
Finland	28892	31387	34381	36207	37058
Sweden	51561	55037	59058	62437	65740
Iceland	1364	1501	1747	2011	2256
Liechtenstein	176	197	228	258	288
Norway	23569	25615	28659	31665	34302
Switzerland	40972	43643	47974	52809	58217
United Kingd	312777	331991	356902	382869	411813
Total	2619328	2761026	2988827	3228856	3464064
