# Supplementary material for: European Prevalence of Polypoidal Choroidal Vasculopathy: A Systematic Review, Meta-Analysis, and Forecasting Study
Source: J Clin Med. 2022 Aug 16;11(16):4766. doi: 10.3390/jcm11164766 (PMC9410106; doi:10.3390/jcm11164766)
Supplement: Supplementary file 1 [file jcm-11-04766-s001.zip › Supplementary File S1.pdf]

Supplementary File S1. Details of the literature search.

PubMed:

History and Search Details

Download
Delete

Search	Actions	Details	Query	Results	Time
#1			Search: **"polypoidal choroidal vasculopathy" AND "macular degeneration" AND (prevalence OR incidence)** Sort by: **Most Recent**<br>"polypoidal choroidal vasculopathy"[All Fields] AND "macular degeneration"[All Fields] AND ("epidemiology"[MeSH Subheading] OR "epidemiology"[All Fields] OR "prevalence"[All Fields] OR "prevalence"[MeSH Terms] OR "prevalance"[All Fields] OR "prevalences"[All Fields] OR "prevalence s"[All Fields] OR "prevalent"[All Fields] OR "prevalently"[All Fields] OR "prevalents"[All Fields] OR ("epidemiology"[MeSH Subheading] OR "epidemiology"[All Fields] OR "incidence"[All Fields] OR "incidence"[MeSH Terms] OR "incidences"[All Fields] OR "incident"[All Fields] OR "incidents"[All Fields]))<br>**Translations**<br>**prevalence:** "epidemiology"[Subheading] OR "epidemiology"[All Fields] OR "prevalence"[All Fields] OR "prevalence"[MeSH Terms] OR "prevalance"[All Fields] OR "prevalences"[All Fields] OR "prevalence's"[All Fields] OR "prevalent"[All Fields] OR "prevalently"[All Fields] OR "prevalents"[All Fields]<br>**incidence:** "epidemiology"[Subheading] OR "epidemiology"[All Fields] OR "incidence"[All Fields] OR "incidence"[MeSH Terms] OR "incidences"[All Fields] OR "incident"[All Fields] OR "incidents"[All Fields]	192	15:46:53

Showing 1 to 1 of 1 entries

EMBASE:

Search History
(10searches found)

Contract

# ▲	Searches	Results	Type	Actions	Annotations
1	"polypoidal choroidal vasculopathy".mp. [mp=title, abstract, heading word, drug trade name, original title, device manufacturer, drug manufacturer, device trade name, keyword heading word, floating subheading word, candidate term word]	2158	Advanced	Display Results More	
2	*polypoidal choroidal vasculopathy/	1059	Advanced	Display Results More	
3	"macular degeneration".mp. [mp=title, abstract, heading word, drug trade name, original title, device manufacturer, drug manufacturer, device trade name, keyword heading word, floating subheading word, candidate term word]	37767	Advanced	Display Results More	
4	*macular degeneration/	1181	Advanced	Display Results More	
5	prevalence.mp. [mp=title, abstract, heading word, drug trade name, original title, device manufacturer, drug manufacturer, device trade name, keyword heading word, floating subheading word, candidate term word]	1243467	Advanced	Display Results More	

6	incidence.mp. [mp=title, abstract, heading word, drug trade name, original title, device manufacturer, drug manufacturer, device trade name, keyword heading word, floating subheading word, candidate term word]	1385001	Advanced	Display Results<br>More	
7	1 or 2	2158	Advanced	Display Results<br>More	
8	3 or 4	37767	Advanced	Display Results<br>More	
9	5 or 6	2471874	Advanced	Display Results<br>More	
10	7 and 8 and 9	188	Advanced	Display Results<br>More	

Combine with:

The Cochrane Library:

1 Cochrane Review matching **"Polypoidal choroidal vasculopathy" in All Text AND "macular degeneration" in Title Abstract Keyword - (Word variations have been searched)**

0 Cochrane Protocols matching **"Polypoidal choroidal vasculopathy" in All Text AND "macular degeneration" in Title Abstract Keyword - (Word variations have been searched)**

60 Trials matching **"Polypoidal choroidal vasculopathy" in All Text AND "macular degeneration" in Title Abstract Keyword - (Word variations have been searched)**

0 Editorials matching **"Polypoidal choroidal vasculopathy" in All Text AND "macular degeneration" in Title Abstract Keyword - (Word variations have been searched)**

0 Special Collections matching **"Polypoidal choroidal vasculopathy" in All Text AND "macular degeneration" in Title Abstract Keyword - (Word variations have been searched)**

0 Clinical Answers matching **"Polypoidal choroidal vasculopathy" in All Text AND "macular degeneration" in Title Abstract Keyword - (Word variations have been searched)**

Web of Science Core Collection, Web of Science Core Collection, BIOSIS Previews, Current Contents Connect, Data Citation Index, Derwent Innovations Index, KCI-Korean Journal Database, and SciELO Citation Index:

Advanced Search > Results for TS=("polypoidal choroidal vasculopathy" AND "macular degene…

303 results from Web of Science Core Collection, BIOSIS Previews, Current Contents Connect, Data Citation Index, Derwent Innovations Index, KCI-Korean Journal Database, SciELO Citation Index:

TS=("polypoidal choroidal vasculopathy" AND "macular degeneration" AND (prevalence OR incidence))
