# Supplementary material for: European Prevalence of Polypoidal Choroidal Vasculopathy: A Systematic Review, Meta-Analysis, and Forecasting Study
Source: J Clin Med. 2022 Aug 16;11(16):4766. doi: 10.3390/jcm11164766 (PMC9410106; doi:10.3390/jcm11164766)
Supplement: Supplementary file 1 [file jcm-11-04766-s001.zip › Supplementary Table S1.pdf]

Supplementary Table S1. Sensitivity analysis of the summary estimate.

Excluded study	Pooled Prevalence	LCI 95%	HCI 95%	Cochran Q	I^2
Ilginis et al. 2012 [31]	8,2%	6,5%	10,1%	4,0	25,1
Ladas et al. 2004 [32]	8,2%	6,3%	10,3%	4,0	25,1
Lorentzen et al. 2018 [10]	9,0%	7,4%	10,8%	0,4	0,0
Scassellati-Sforzolini et al. 2001 [33]	8,0%	6,4%	9,7%	3,2	6,4
Yadav et al. 2017 [34]	7,7%	6,0%	9,7%	3,1	4,6
