# Supplementary figures and images for: European Prevalence of Polypoidal Choroidal Vasculopathy: A Systematic Review, Meta-Analysis, and Forecasting Study
Source: J Clin Med. 2022 Aug 16;11(16):4766. doi: 10.3390/jcm11164766 (PMC9410106; doi:10.3390/jcm11164766)

**Supplementary Figure S1.** Funnel plot for the evaluation of risk of bias across studies.

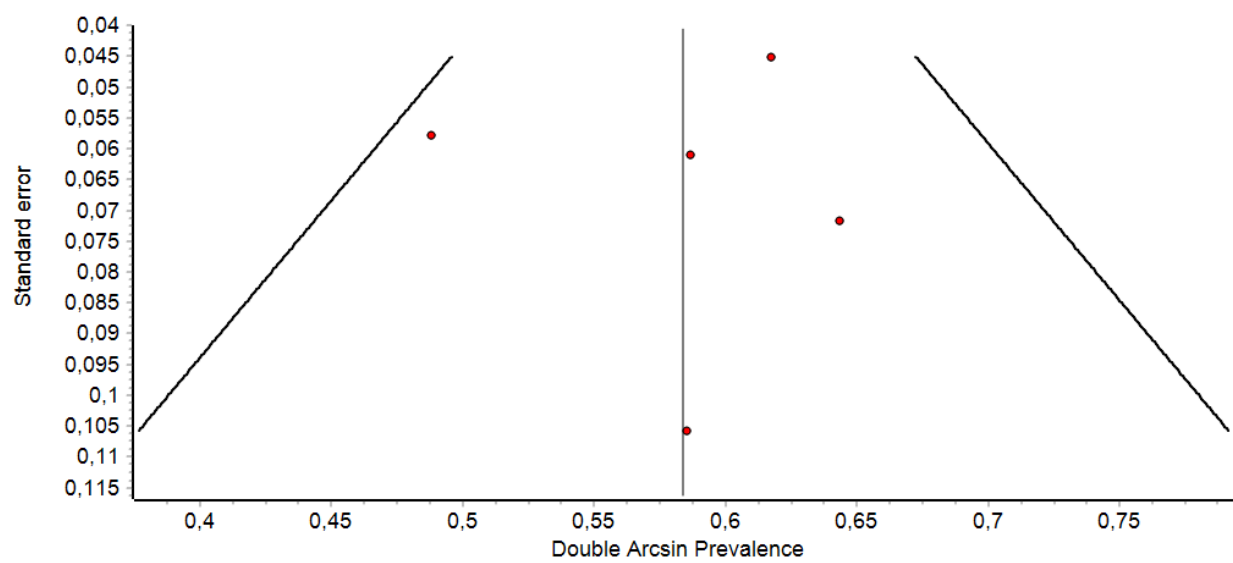

Supplement: Supplementary file 1 [file jcm-11-04766-s001.zip › Supplementary Figure S1.pdf]
